# Evaluation of Preference and Utility Measures for Transoral Thyroidectomy

**DOI:** 10.1177/00034894221094950

**Published:** 2022-05-03

**Authors:** Vincent Wu, Shireen Samargandy, Justine Philteos, Jesse D. Pasternak, John R. de Almeida, Eric Monteiro

**Affiliations:** 1 Department of Otolaryngology—Head & Neck Surgery, Sinai Health, University of Toronto, Toronto, ON, Canada; 2 Department of Surgery, University Health Network, University of Toronto, Toronto, ON, Canada; 3 Institute for Health Policy Management and Evaluation, University of Toronto, Toronto, ON, Canada

**Keywords:** thyroid, thyroidectomy, transoral, utility, standard gamble, preference

## Abstract

**Background:**

Traditional, trans-cervical thyroidectomy results in the presence of a neck scar, which has been shown to correlate with lower quality of life and lower patient satisfaction. Transoral thyroid surgery (TOTS) has been utilized as an alternative approach to avoid a cutaneous incision and scar by accessing the neck and thyroid through the oral cavity. This study was designed to evaluate patient preference through health-state utility scores for TOTS as compared to conventional trans-cervical thyroidectomy.

**Methods:**

In this cross-sectional study, patient preferences were elicited for TOTS and trans-cervical thyroidectomy with the use of an online survey. Respondents were asked to consider 4 hypothetical health scenarios involving thyroid surgery with varying approaches. Health-state utility scores were elicited using visual analog scale and standard gamble exercises.

**Results:**

Overall, 516 respondents completed the survey, of whom 261 (50.6%) were included for analysis, with a mean age of 41.5 years (SD 14.9 years), including 171 (65.5%) females. Health utility scores were similar for TOTS and conventional transcervical techniques. Statistically significant differences in the standard gamble utility score were noted for gender and ethnicity across all scenarios. Comparisons of visual analog score utilities were not statistically significant based on respondent demographics.

**Conclusion:**

Preferences for TOTS and trans-cervical thyroidectomy did not significantly differ in the current study. Females and white ethnicity indicated stronger preference for a TOTs approach compared to males and other ethnicities, respectively. Some literature suggests certain types of patients who might prefer minimally invasive thyroidectomy more so than other patients—in keeping with the current findings of this study.

## Introduction

Thyroid surgery has evolved considerably over the past centuries, with significant improvements in surgical technique corresponding to improvements in complications and morbidity.^1^ The transcervical approach for thyroidectomy is the most widely used approach, which utilizes a low transverse anterior neck incision. It is preferred by some surgeons for the ease of access it provides in order to safely execute the critical part of the procedure, namely dissection of the recurrent laryngeal nerve. The downside of this approach is the visible scar.^2^ The presence of a neck scar has been shown to correlate with a lower quality of life and lower patient satisfaction—irrespective of how well it heals.^3^ As such, many surgeons have championed extra-cervical approaches in order to improve the cosmetic outcome by placing the scar in a less conspicuous location.^4,5^

Various extra-cervical approaches have been described in the literature, including endoscopic or robotic trans-axillary, axillo-breast, retro-auricular, and transoral approaches.^6,7^ While the majority of these extra-cervical thyroidectomy approaches simply relocate the cervical scar to a concealed location, transoral thyroid surgery (TOTS) has recently gained favor as it avoids a cutaneous incision altogether by accessing the thyroid through a gingivobuccal incision in the oral cavity.^8-10^ Interestingly, the literature has demonstrated that patients may prefer scarless surgery—even with increased procedural risk of mental nerve palsy.^11-14^

Clinical decision analysis is a methodology where empirical data, including patient preferences, are quantified and integrated into the medical decision making process.^15^ The preferred method for evaluating patients’ treatment perspectives comes from patient-derived health-state utilities, as this allows for the exploration of trade-off preferences based on risk and benefits of different treatment options.^16,17^ The standard gamble technique elicits a respondent’s preference for a particular health state (eg, thyroidectomy with or without a cervical scar), by asking the respondent to choose between the proposed health state and an alternate scenario that has a chance of either achieving perfect health or conversely a risk of death. By progressively and iteratively altering the risk of death associated with the alternate scenario that provides perfect health until the respondent changes their response between the 2 choices, one may determine the utility for that health state. Utility is measured on a scale of 0 to 1, where 0 equals death and 1 equals perfect life.^18^ Herein, we aimed to evaluate patient preference through health-state utility scores for TOTS as compared to conventional thyroidectomy for thyroid cancer.

## Methods

### Study Design

This was a cross-sectional study, designed to elicit participant preferences for TOTS and conventional trans-cervical thyroidectomy with the use of an online survey. This study utilized hypothetical health scenarios where thyroid surgery was necessary. Respondents were recruited from Amazon Mechanical Turk (MTurk), an online service that facilitates the completion of tasks or surveys posted on the platform by registered users. MTurk has been used in similar study designs and has been shown to be comparable to conventional data collection methods.^19-22^ This study was approved by the Sinai Health Research Ethics Board #19-0132-E.

### Study Population

Adult participants from the United States and Canada were recruited through MTurk. Participants were excluded if under the age of 18, or if they have had previous head and neck surgery including thyroidectomy, or previous head and neck radiation. Moreover, participants were excluded if they had failed the attention-check questions, or incorrectly completed the standard gamble screening exercise. Selections based on age (≥40 years old, <40 years old) and gender were made, as both were previously identified factors that influenced preferences in thyroidectomy.^13,23^

### Intervention

Participants were asked to read 4 hypothetical clinical scenarios outlining different health states and treatment options (Supplemental 1). Standard gamble exercises were performed for each scenario (Supplemental 2). Participants were also asked to place a mark on a 10-point visual analog scale reflecting how they perceive the treatment-based scenarios relative to death (0) and full health (10). Participants also completed an 8-item survey, capturing demographic information (Supplemental 3). Three attention check questions were imbedded within the survey, similar to other studies utilizing MTurk.^20,24^

### Scenario Design

Clinical scenarios were used to outline the benefits and risks associated with TOTS and conventional trans-cervical thyroidectomy. Four hypothetical scenarios were created: 2 for total thyroidectomy and 2 for hemithyroidectomy with both surgical approaches offered (Supplemental 1). For the total thyroidectomy scenarios, participants were presented with a diagnosis of multi-focal thyroid cancer without nodal disease. For the hemithyroidectomy scenarios, participants were asked to imagine they had an isolated thyroid cancer nodule only involving 1 thyroid lobe without nodal disease. The presence or absence of a visible surgical scar, length of surgery, length of hospital stay, and specific risks associated with the surgery were noted. Sources of information used for generating the clinical scenarios stemmed from large clinical studies on effectiveness and safety profiles of TOTS and trans-cervical thyroidectomy.^2,25^ The vignettes were vetted for face validity by 3 high-volume thyroid surgeons (JDA, JP, and EM).

### Statistical Analysis

SPSS (v20, International Business Machine, United States) was used for all statistical analyses. Statistical significance was set to P < .05. Data were inputted into a spreadsheet designed specifically for the study. Descriptive statistics were used to display the baseline demographics of the participants. Utility scores derived from the visual analog scale and standard gamble were presented as means and standard deviation (SD). Parametric tests, such as 1-way analysis was variance with Bonferroni’s correction, were used for visual analog scale utilities, as it was normally distributed. Non-parametric tests, including Kruskal-Wallis test with post-hoc Dunn’s test, were used for standard gamble utilities. Paired *t*-tests were used to evaluate patient preferences from generated utility scores based on results of the visual analog scale and standard gamble exercises.

## Results

**Figure 1.**
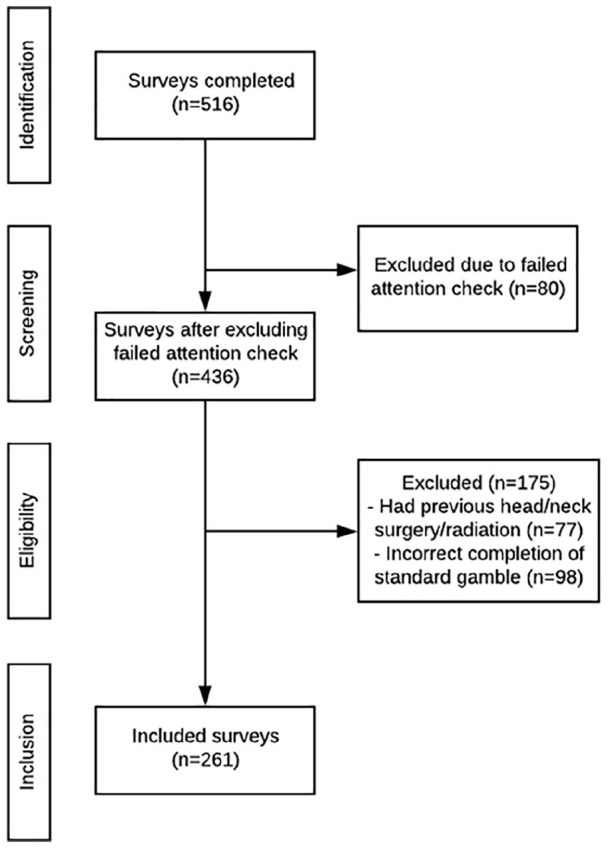
Participant survey inclusion flow-chart.

A total of 516 individuals completed the survey. Eighty participants failed the attention check question and were consequently excluded. Of the remaining participants, 77 were excluded according to our outlined exclusion criteria and another 98 did not perform the standard gamble exercise correctly and their responses were removed (ie, answering “yes” to higher degree of risk, after answering “no”), as outlined in Figure 1. Thus, 261 (50.6%) participants were included in the final analysis. Included participant demographics are described in Table 1. The majority of participants were self-identified white (77.4%), female (65.5%), and had an annual income less than $50 000 CDN (49.8%).

**Table 1.** Participant Demographics.

Characteristics	N = 261	Percentage (%)
Age		
18-39	151	57.9
>40	110	42.1
Sex		
Female	171	65.5
Male	87	33.3
Other	3	1.1
Marital status		
Married/divorced/widowed	169	64.8
Single/never married	92	35.2
Ethnicity		
Asian	13	5.0
Black	15	5.7
Hispanic	15	5.1
Native/Indian	16	6.1
White	202	77.4
Highest level education		
High school	44	16.9
College/University	154	59.0
Post-graduate	63	24.1
Annual income ($ CDN)		
<$50 000	130	49.8
$50 000-$100 000	88	33.7
>$100 000	43	16.5
Perceived health		
Excellent	27	10.3
Very good	79	30.3
Good	106	40.6
Fair	42	16.1
Poor	7	2.7

### Surgical Technique Preferred

The first 2 scenarios asked participants to identify which surgical technique they would prefer for total thyroidectomy and hemithyroidectomy. For total thyroidectomy, 132 (50.6%) respondents preferred TOTS and 129 (49.4%) trans-cervical thyroidectomy. For hemi-thyroidectomy, 125 (47.9%) had a preference for TOTS, and 136 (52.1%) answered transcervical thyroidectomy. There were no statistically significant differences in the surgical technique preference when comparing total thyroidectomy with hemithyroidectomy (P = .54).

### Utility Scores

The utility scores for the visual analog scale and standard gamble are outlined in Table 2. As noted, there were no statistically significant differences in comparing TOTS versus transcervical approaches across the scenarios. We then conducted a secondary analysis with regards to standard gamble scores and participant demographics. We noted statistically significant differences for standard gamble scores based on participant gender and ethnicity. With regards to gender, we noted statistically significant higher utility scores across the 4 scenarios for female participants as outlined in Table 3. With regards to ethnicity, comparison of differences in standard gamble utilities between white and non-white ethnicity groups were performed. Participants identifying as “white” had statistically significant higher utility scores across the 4 scenarios which is also noted in Table 3.

**Table 2.** Healthy-State Utility Scores Across the 4 Hypothetical Scenarios Presented to Participants.

Scenarios	TOTS approach	Transcervical approach	*P*-value
Total thyroidectomy			
Visual analog scale	0.54 (0.25)	0.55 (0.24)	.801
Standard gamble	0.93 (0.15)	0.94 (0.14)	.372
Hemi-thyroidectomy			
Visual analog scale	0.55 (0.24)	0.55 (0.24)	.535
Standard gamble	0.94 (0.15)	0.94 (0.14)	.250

*Note.* This table notes the visual analog scores and standard gamble utility scores for the 4 hypothetical scenarios. The utility scores are documented as means (SD).

Abbreviation: TOTS, transoral thyroid surgery.

**Table 3.** Standard Gamble Scores Based on Gender and Ethnicity.

Scenarios	Gender			Ethnicity		
	Male	Female	*P*-value	White	Non-White	*P*-value
	Mean ± SD	Mean ± SD		Mean ± SD	Mean ± SD	
Total thyroidectomy						
TOTS	0.92 ± 0.17	0.94 ± 0.15	*P* = .0183	0.95 ± 0.13	0.88 ± 0.21	.0127
Transcervical	0.93 ± 0.14	0.94 ± 0.14	*P* = .0110	0.95 ± 0.13	0.90 ± 0.18	.0204
Hemi-thyroidectomy						
TOTS	0.93 ± 0.15	0.94 ± 0.14	*P* = .0399	0.95 ± 0.13	0.89 ± 0.19	.0076
Transcervical	0.93 ± 0.15	0.94 ± 0.14	*P* = .0133	0.95 ± 0.12	0.90 ± 0.19	.0090

*Note.* Standard gamble results were compared based on respondent demographics. Statistically significant differences were noted in the standard gamble utility score for gender and ethnicity across all 4 scenarios. The standard gamble scores are reported as mean ± SD. The *P*-value demonstrating significance in the difference in scores across the scenarios is also noted.

## Discussion

In this study, we aimed to evaluate patient preference through health-state utility scores for TOTS and conventional transcervical thyroidectomy for thyroid cancer. Our data suggests that, for North Americans, participants did not demonstrate a strong preference for transoral thyroidectomy over a transcervical approach. Furthermore, the findings suggest that utility may be impacted by gender and ethnicity—with females and white participants indicating higher standard gamble utility scores when compared to males and non-white participants.

The literature on patient preference with regards to thyroidectomy scar is varied. Similar to the findings of our current study, previous literature has demonstrated that the majority of patients are not bothered by a thyroidectomy scar.^23,26,27^ Interestingly, these findings are not in keeping with another study exploring patient preferences for thyroid surgery utilizing extra-cervical approaches. Coorough et al^13^ explored patient preferences for trans-axillary thyroidectomy versus traditional transcervical thyroidectomy via survey. They concluded that participants with a preference for trans-axillary approach—avoiding a conventional transcervical scar—were younger, and female. Interestingly, a recent study exploring patient satisfaction with post-thyroidectomy scars concluded that the mean scar rating of patients with a diagnosis of cancer was shown to be far worse than those with benign disease. It is hypothesized that the patients’ psyche post-cancer diagnosis may have a negative impact upon their scar satisfaction.^14^

A recent study, employing an eye-tracking software, found that the presence of a visible neck scar leads to significant gaze distraction of viewers away from the face.^28^ Furthermore, a study exploring third party rater preference for thyroidectomy scar noted that raters preferred low, short scars; as opposed to, longer thyroidectomy scars.^29^ Interestingly, a recent study utilized a discrete choice experiment to evaluate the influence of cosmetic concerns on patients’ decision-making processes when choosing among different thyroidectomy approaches. They similarly concluded that presence of a neck scar also influenced patient decision-making, but this was dependent on patient age with younger patients preferring a scarless approach.^30^ This has important implications in the current study as the majority of respondents were under the age of 39 which may have skewed the preference for a scarless approach. Nonetheless, this has very interesting and complex applications for the primary surgeon. With regards to the decision making process on selecting the appropriate surgical treatment, discussions should be individualized to each patient as surgeons cannot broadly assume all patients will choose the scarless procedure. Gutknecht et al^31^ reported that patients do not fully comprehend the concept of a complication rate before surgery. This explains why some patients may be easily persuaded to agree to a procedure that offers improved cosmesis, while potentially harboring an increased rate of complications. A recent retrospective study from Brazil assessed complication rates and learning curves for TOTS thyroidectomy and traditional transcervical. They concluded that in high-volume centers the complication rates were comparable. However, the operative time for TOTs was significantly longer and there are complications unique to a TOTs approach (eg, injury to the mental nerves).^32^ Perrier et al published guiding principles as part of A New Technology Task Force, that served as a framework for the safe implementation of emerging technologies in thyroid surgery. The authors noted that the final decision to pursue non-conventional thyroidectomy should be left up to the surgeon and the patient.^33^ These are important concepts to consider in guiding the conversation with patients with regards to treatment decisions.

We noted heterogeneity with regards to health utility scores based on the method by which they were derived. In our study, both the standard gamble and visual analog scale were employed to capture preference for TOTS and transcervical thyroidectomy across hypothetical health states. While we found significant differences with respect to gender and ethnicity in utilities scores derived from standard gamble, there were no noted differences in the utility based on visual analog scale across the scenarios. Thus, with respect to obtaining estimates of health state utility, the standard gamble and visual analog scale measures yielded utility estimates that are comparable for decision-making purposes. Furthermore, across all scenarios, visual analog scores were lower than the standard gamble scores. This is further demonstrated in the Neumann–Morgenstern utility theory which supports the accuracy of standard gamble given the trade-off aspect which is not found in the visual analog scales. This is a well-recognized phenomenon, likely due to a combination of respondents’ risk aversion and biased risk perception.^16,34,35^ The standard gamble exercise involves inherent risk-taking, as a participant’s decision involves risk; whereas, this does not hold true for visual analog scales. The biased risk perception phenomenon describes an overestimation of small percentages. Although the visual analog scale is more convenient to administer and easier to comprehend for participants, the drawbacks include its lack of risk trade-off inclusion. Therefore, visual analog scales are not based on expected-utility theory; and as a result, the utility scores tend to be lower. Although there is no preferred methodology for deriving utility scores, utilities which are derived by standard gamble reliably resemble the foundations from which cost-effectiveness analysis is based.^16^ This explains the differences with regards to utility scores as captured through the standard gamble exercise and with the visual analog scale in our study.

The limitations of this study are inherent to survey-based studies. With regards to the use of MTurk as a survey delivery platform, attention check questions were built in to exclude individuals not actively responding. There was a large proportion of individuals who failed the attention check, which may have introduced selection bias in our included data population. Furthermore, the average age of respondents is younger than the average age of patients undergoing a thyroidectomy. Additionally, it is possible that the wording used within the health scenarios may have affected the utility assessment. We attempted to minimize this by involving multiple thyroid surgery experts in the creation and development of the scenarios, ensuring its comprehensiveness by the lay-public. Furthermore, we note that females and participants identifying as white had statistically significant higher standard gamble scores in general across procedure types which limits the utility of the conclusions one draw. Lastly, the cognitive complexity of decision-making processes are not entirely captured through health utility scores.

## Conclusion

In evaluating patient preference through health-state utility scores for TOTS as compared to conventional thyroidectomy for thyroid cancer, we noted that preferences for surgical approach were not significantly different across the 4 hypothetical health scenarios. Utility, as assessed through standard gamble, were overall high when compared to visual analog scores. Furthermore, it was noted that utility scores were impacted by gender and ethnicity—with higher scores seen in those who identified as female and white ethnicity.

## Supplemental Material

sj-docx-1-aor-10.1177_00034894221094950 – Supplemental material for Evaluation of Preference and Utility Measures for Transoral Thyroidectomy Click here for additional data file. Supplemental material, sj-docx-1-aor-10.1177_00034894221094950 for Evaluation of Preference and Utility Measures for Transoral Thyroidectomy by Vincent Wu, Shireen Samargandy, Justine Philteos, Jesse D. Pasternak, John R. de Almeida and Eric Monteiro in Annals of Otology, Rhinology & Laryngology
