# Supplementary material for: Evaluation of Preference and Utility Measures for Transoral Thyroidectomy
Source: Ann Otol Rhinol Laryngol. 2022 May 3;132(4):381–6. doi: 10.1177/00034894221094950 (PMC9989232; doi:10.1177/00034894221094950)
Supplement: sj-docx-1-aor-10.1177_00034894221094950 – Supplemental material for Evaluation of Preference and Utility Measures for Transoral Thyroidectomy [file sj-docx-1-aor-10.1177_00034894221094950.docx]

**APPENDIX**

Appendix 1: Hypothetical Scenarios Presented to Participants

**Scenario # 1**

The thyroid is a gland in front of your neck responsible for regulating bodily functions.

Imagine a doctor found two lumps in your thyroid gland. They were sampled and found to be cancer. You are offered the conventional transcervical surgery approach to remove the entire thyroid gland, with a success rate of 99% to provide a complete cure.

The conventional transcervical surgery involves an incision/cut made through the front of your neck to take out the thyroid gland. During the surgery, you will be asleep under general anesthesia and the surgery will take approximately 1 hour 30 minutes. You will be in hospital for 1-2 days after the surgery and then sent home.

Surgical Risks - You may experience the following:

- Neck scar 100% (You will have a scar approximately 2 inches)
- Nerve injury leading to hoarse/breathy voice or choking (temporary and/or permanent) ~1%
- Low calcium requiring calcium pills (temporary ~20%, permanent ~2%)
- Bleeding - life threatening requiring a second surgery <1%
- Infection <1%

The black dotted line represents your incision and scar:


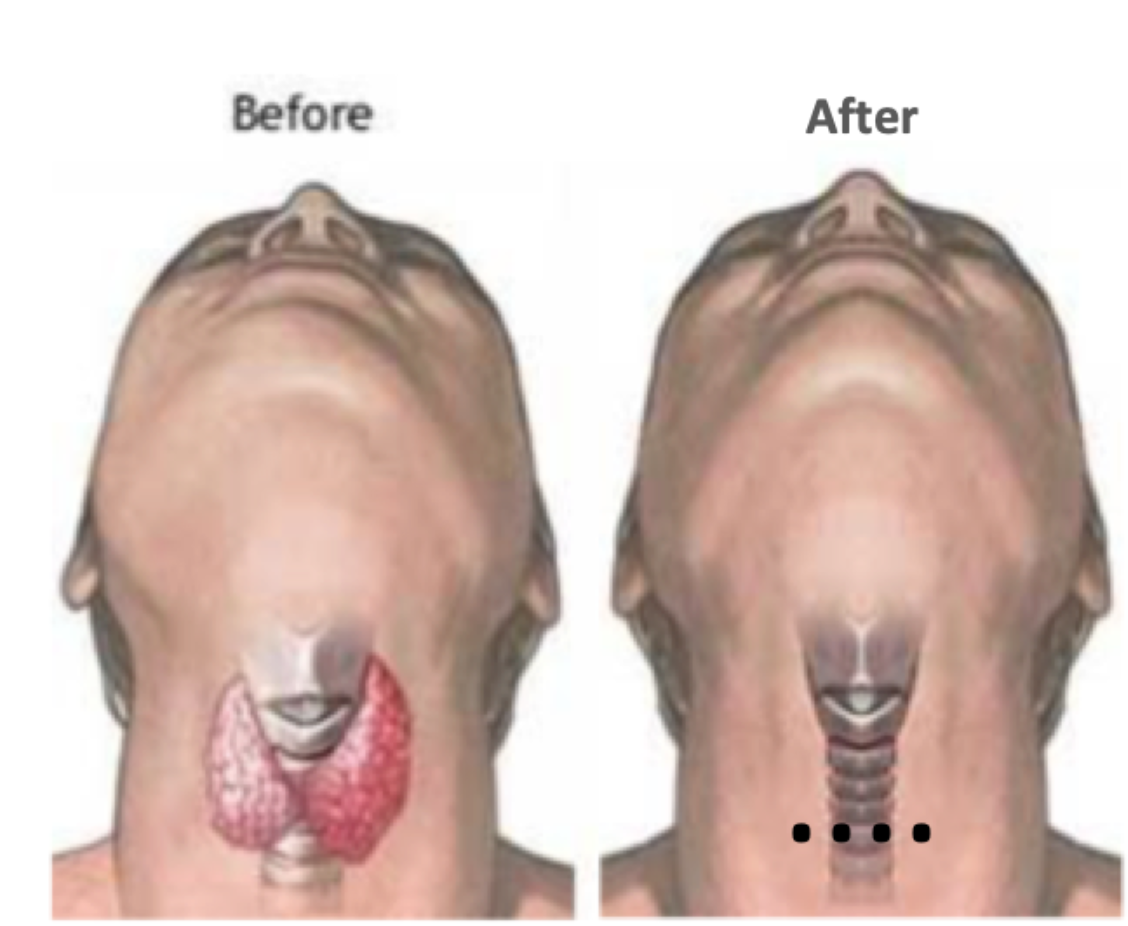


**Scenario #2**

The thyroid is a gland in front of your neck responsible for regulating bodily functions.

Imagine a doctor found two lumps in your thyroid gland. They were sampled and found to be thyroid cancer. You are offered the new transoral surgery approach to remove the entire thyroid gland, with a success rate of 99% to provide a complete cure.

The transoral surgery involves an incision/cut made inside your mouth to take out the thyroid gland. During the surgery, you will be asleep under general anesthesia and the surgery will take approximately 2.5 hours. You will be in hospital for 1-2 days after the surgery and then sent home.

Surgical Risks - You may experience the following:

- Conversion to conventional (transcervical) thyroidectomy ~1% (if surgery through the mouth fails, requiring surgery through a neck incision)
- Neck scar (if conversion to conventional thyroidectomy) ~1%
- Nerve injury leading to hoarse/breathy voice or choking (temporary and/or permanent) ~5%
- Low calcium requiring calcium pills, (temporary ~20%, permanent ~2%)
- Bleeding - life threatening requiring a second surgery <1%
- Infection <1%
- Chin numbness (temporary and/or permanent) ~1%
- Injury to the lips and surrounding skin ~1%


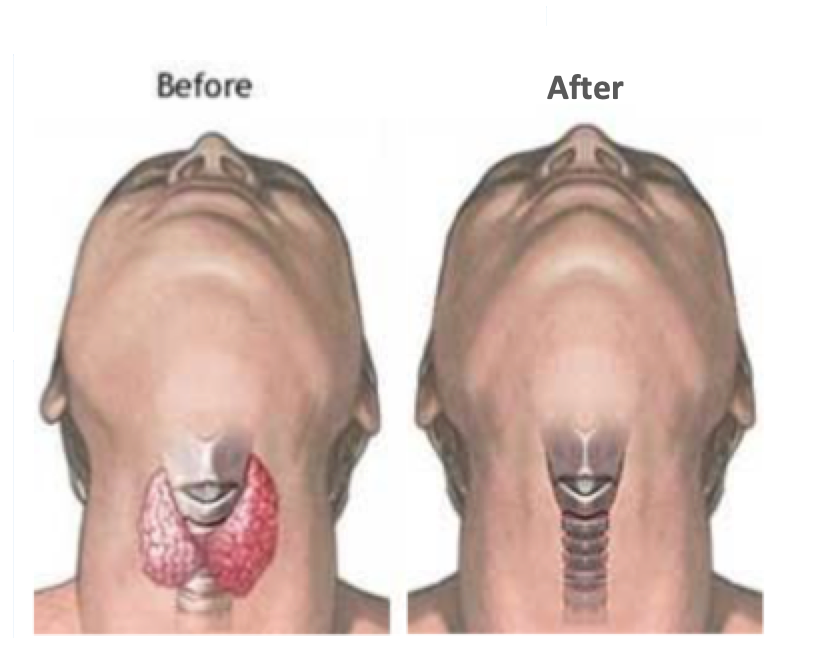


**Scenario #3**

The thyroid is a gland in front of your neck responsible for regulating bodily functions.

Imagine a doctor found a lump on one side of your thyroid gland. It was sampled and found to be thyroid cancer. You are offered the conventional transcervical surgery approach to remove half of the thyroid gland, with a success rate of 99% to provide a complete cure.

The conventional transcervical surgery involves an incision/cut made through the front of your neck to take out half of the thyroid gland. During the surgery, you will be asleep under general anesthesia and the surgery will take approximately 1 hour. You will be in hospital for 1-2 days after the surgery and then sent home.

Surgical Risks - You may experience the following:

- Neck scar 100% (You will have a scar approximately 1.5 inches)
- Nerve injury leading to hoarse/breathy voice or choking (temporary and/or permanent) ~1%
- Bleeding - life threatening requiring a second surgery <1%
- Infection <1%

The black dotted line represents your incision and scar:


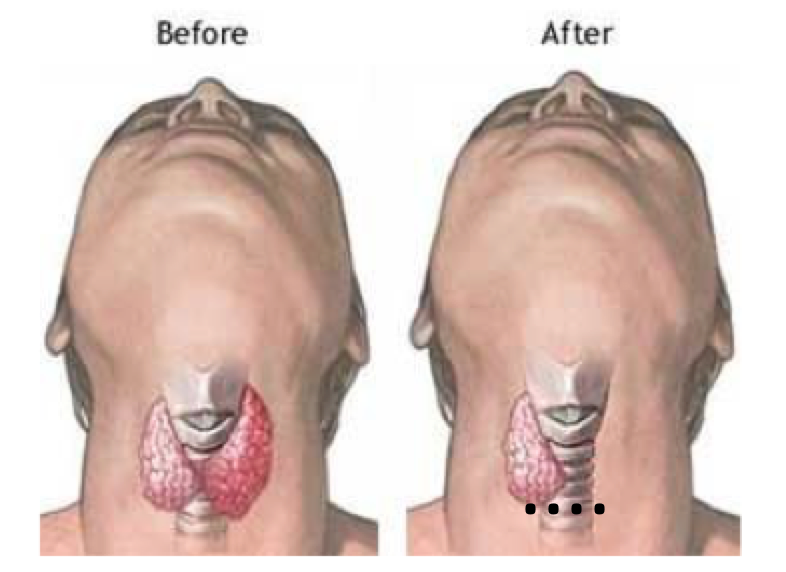


**Scenario #4**

The thyroid is a gland in front of your neck responsible for regulating bodily functions.

Imagine a doctor found a lump on one side of your thyroid gland. It was sampled and found to be thyroid cancer. You are offered the new transoral surgery approach to remove half of the thyroid gland, with a success rate of 99% to provide a complete cure.

The transoral surgery involves an incision/cut made inside the mouth to take out half of the thyroid gland. During the surgery, you will be asleep under general anesthesia and the surgery will take approximately 1 hour and 45 minutes. You will be in hospital for 1-2 days after the surgery and then sent home.

Surgical Risks - You may experience the following:

- Conversion to conventional (transcervical) thyroidectomy ~1% (if surgery through the mouth fails, requiring surgery through a neck incision)
- Neck scar (if conversion to conventional thyroidectomy) ~1%
- Nerve injury leading to hoarse/breathy voice or choking (temporary and/or permanent) ~5%
- Bleeding - life threatening requiring a second surgery <1%
- Infection <1%
- Chin numbness (temporary and/or permanent) ~1%
- Injury to the lips and surrounding skin ~1%


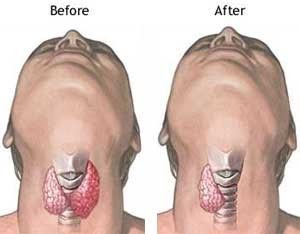


Appendix 2: Standard Gamble Exercise Example

Below is a thought experiment for transoral thyroidectomy used to determine how you feel about the described scenario.

You can choose the outlined treatment - transoral hemi-thyroidectomy

OR

You can take a single pill that has the following features:

- It has the same benefit as the surgery

- It does not change the risk of the thyroid cancer returning or dying from the cancer

- It does not have the side effects of surgery nor do you require hospitalization

Taking the pill however, has a risk of sudden painless death in your sleep.

Example of standard gamble exercise:


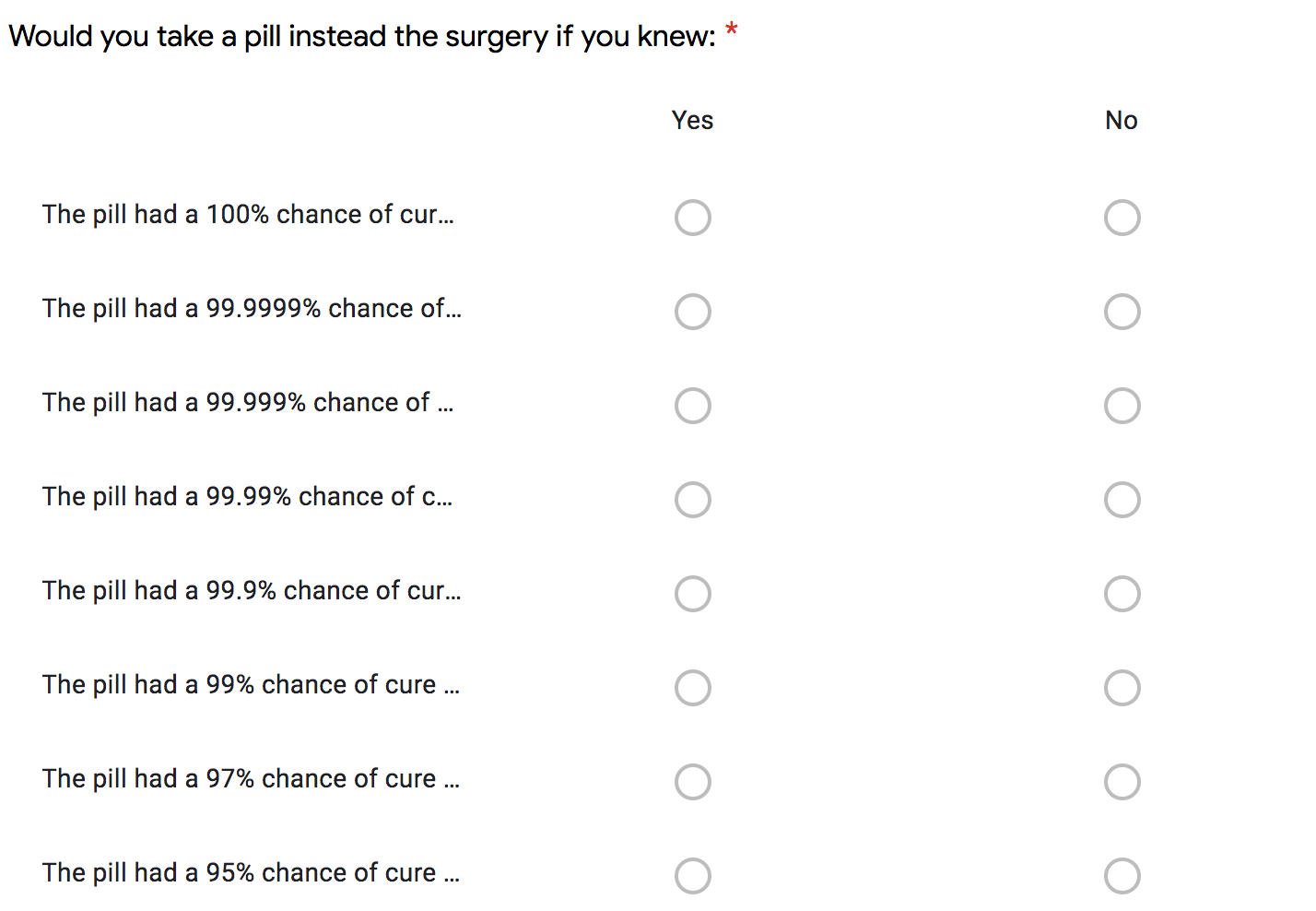


Appendix 3: Demographic Questionnaire

1. What is your identified gender:

a. male

b. female

c. prefer to not disclose

d: Other: ______

2. What is your age? ______

3. What is your current marital status?

a. Single/Never Married

b. Married/Divorced/Widowed

c. Other: ______

4. What is your ethnicity?

a. African-American/Canadian

b. Asian/Oriental/Pacific Islander

c. Caucasian

d. Hispanic

e. Indian/South Asian

f. Native-American/Canadian

g. Other:

5. What is your highest level of education?

a. High-school or below

b. College/University

c. Post-graduate studies

6. What is your annual household income?

a. <$50,000 (CDN)

b. $50,000 to $100,000 (CDN)

c. >$100,000 (CDN)

7. How would you rate your overall health over the last year?

a. Excellent

b. Very good

c. Good

d. Fair

e. Poor

8. Have you previously had surgery or radiation therapy to the neck?

a. Yes

b. No

c. If yes, please specify the type of surgery or radiation therapy that you had to the neck: ________
